# When Control Exacerbates Distress: A Qualitative Study Exploring the Experiences of Hong Kong Chinese Parents in Caring for a Child with Asthma

**DOI:** 10.3390/ijerph15071372

**Published:** 2018-06-30

**Authors:** Yuen-yu Chong, Doris Leung, Yim-wah Mak

**Affiliations:** 1 School of Nursing, The Hong Kong Polytechnic University, Hong Kong Special Administrative Region, Hong Kong, China; connie.yy.chong@connect.polyu.hk (Y.C.); doris.leung@polyu.edu.hk (D.L.); 2 The Lawrence S. Bloomberg Faculty of Nursing, University of Toronto, 155 College Street, Suite 130, Toronto, ON M5T 1P8, Canada

**Keywords:** parents, children, asthma, qualitative research, psychological distress, psychological adjustment

## Abstract

*Background*: Many parents have difficulty managing childhood asthma. In Hong Kong (HK), while medication is the primary form of treatment, traditional Chinese medicine is another favored option. In addition, HK follows a dual-track healthcare system, which may pose unique experiences for Chinese parents in managing childhood asthma. This qualitative descriptive study aimed to explore the experiences of HK Chinese parents in caring for their children with asthma. *Methods*: Fourteen HK Chinese mothers of children (aged 3–10) suffering from asthma were purposively sampled to participate in individual, semi-structured interviews. A realist approach following conventional content analysis was used to interpret the interviews. *Results*: The mothers expressed feelings of uncertainty, fear of asthma crises, and searched for ways to cope. As long as their child’s asthma symptoms recurred, the mothers’ distress continued. Their distress was sometimes exacerbated by self-doubt and worries over whether they would receive adequate support from their family and healthcare professionals. *Conclusions*: Helping parents to understand their limits may help them be more open to varied aspects of their caregiving experiences, and thus to cope better. Psychological interventions together with traditional educational training may help to alleviate the psychological difficulties of parents.

## 1. Introduction

Asthma is the most common chronic respiratory disease, affecting over 235 million people worldwide [1]. The International Study of Asthma and Allergies in Childhood (ISACC), involving approximately 1.2 million schoolchildren worldwide, reported a global prevalence of asthma of 11.7% for children aged 6–7 years, and 14.1% for children aged 13–14 years, respectively [2]. Globally, as countries industrialize and more people adopt Western lifestyles, the prevalence of childhood asthma has grown by approximately 50% each decade. In China, the growth is even more pronounced [3], particularly in Hong Kong (HK), where the highest rate of prevalence, at 10.2%, occurs in children. This figure is higher than that of other urban cities such as Beijing (6.3%) and Guangzhou (6.9%) [4].

Managing childhood asthma can be challenging for many parents. In particular, the unpredictability and life-threatening nature of asthma can impose a heavy psychological burden on parents [5]. During the pre-diagnostic phase, parents often report feelings of uncertainty and powerlessness [6]. In their attempts to control childhood asthma, parents engage in a variety of asthma management activities, including compliance with medical treatments [7], monitoring their child’s asthma symptoms, and coordinating family routines to avoid environmental triggers [8,9,10]. Generally, when compared with parents of healthy children, parents of children with asthma experience a greater level of stress [11], anxiety, and depressive symptoms [12].

Asthma is regarded as effectively treatable when parents and their children are educated about how to manage the disease and have access to high-quality healthcare services [1]. Yet, even after gaining access to the healthcare system, and obtaining a confirmed a diagnosis of their child’s asthma [6,13], rather than feeling relieved, some parents report a continued sense of fear and anxiety that their child’s asthma may become life-threatening [14,15,16].

In HK, the healthcare system runs on a dual-track basis. Children who have been diagnosed with asthma receive asthma-related healthcare services on a fee-for-service basis in private clinics and hospitals, and/or on a subsidized basis in general outpatient clinics and public hospitals run by the HK Hospital Authority [17]. Parents usually need to decide which of the abovementioned healthcare services to choose when their child has an acute asthma attack or needs follow-up care.

Following the best evidence from the Global Initiative for Asthma, the primary treatment of childhood asthma is medication for controlling asthma symptoms, including inhaled corticosteroids and short-acting bronchodilators [1]. In HK, traditional Chinese medicine is another option favored by the local population. The perception of traditional Chinese medicine is that it can cure the root of the problem [18]. Hence, given the social structures of a dual-track healthcare system and HK Chinese parents’ perceptions of the problem, these parents may have unique experiences of caring for a child with asthma. As information on the state of asthma management among families in the Asia-Pacific region is limited, this study was conducted to explore the experiences of HK Chinese parents in caring for a child with asthma.

## 2. Methods

### 2.1. Study Design and Participants

A qualitative descriptive design was chosen because it is appropriate for examining the experiences of individuals situated in a particular context who have received little attention in the literature [19]. A realist paradigm was employed to explore how individuals experience events as they occur and in the ways that they occur; that is, to determine what social structures (e.g., cultural, familial, or institutional) trigger actions [20].

The study took place at one Ambulatory Care Centre (ACC) under the Department of Pediatric and Adolescent Medicine in a public hospital in Hong Kong. The ACC is a specialist outpatient clinic where children aged 18 years or below with a diagnosis of chronic respiratory disease, such as asthma, allergic rhinitis, pneumonia, and obstructive sleep apnea, can access medical consultation services provided by pediatricians, and receive education from an Advanced Practice Nurse. The majority of children with asthma, who were under the care of the ACC, attended the emergency department at least once due to an asthma attack over the past six months.

Parents were eligible to participate in the study if they fulfilled the following criteria: (a) were either a father or a mother (age 18–65 years) who was the primary caregiver of their child, (b) had a child aged three to 12 years who had been diagnosed with asthma by a physician (International Classification Diseases—10 codes J45, J46), (c) were HK permanent residents, (d) lived together with their child with asthma, and (e) able to communicate in Cantonese. Parents whose child with asthma had mental and/or congenital problems were excluded. To seek phenomenal variation, we followed the recommendation of Sandelowski, who suggested the use of one kind of purposive sampling, known as criterion sampling [21]. Criterion sampling refers to the recruitment of specific individuals who researchers expect from the literature or from clinical experience will have varying experiences of the phenomenon. In our study, fathers were expected to have different perceptions of their caregiving than mothers.

### 2.2. Data Collection

Parents were invited to complete a short survey to indicate their socio-demographic characteristics, personal and family history of asthma, and their children’s clinical characteristics. Next, data were collected through individual face-to-face interviews in Cantonese. A semi-structured interview guide containing the following open-ended questions was used: (a) “I wish you would share your experiences in taking care of a child with asthma. What experiences do you think are most worthy of sharing? Which were the most memorable to you? And how did you feel at the time?” (b) “As a parent of a child with asthma, what do you do to take care of your child?” (c) “What are the most challenging situations you have faced when taking care of a child with asthma?” (d) “What are the issues of most concern to you when taking care of a child with asthma?” This interview guide was designed by the research team and refined after a pilot test involving two parents of children with asthma. Prompts were used where necessary to encourage more detailed responses, such as, “Can you describe this in a bit more detail?” and “Please tell me more about …”

Interviews were conducted by the first author, who is a registered nurse with experience in pediatric care and who had no affiliation to any of the participants prior to the study. Interviews were held in a private room of the ACC at a time that was convenient to the participants, and audio-recorded with the participants’ permission. Field notes were taken to capture non-verbal cues within 24 h of each interview. Each interview lasted between 45 to 75 min. Data collection continued until the research team determined that data saturation had been reached after 14 interviews. As suggested by Sandelowski, an adequate sample is not determined by the number of participants, but rather, by the adequacy of the sampling strategy to get “information-rich cases” to achieve saturation of patterns [21]. Indeed, Sandelowski [21] suggests that ten descriptions of a target experience may be judged adequate for certain kinds of homogenous or critical case sampling [21].

### 2.3. Ethical Considerations

Before the commencement of the study, ethical approval for this study was obtained from the New Territories West Cluster Clinical Research Ethics Committee (reference number: NTWC/CREC/15042) and from the Human Subjects Ethics Application Review System of the Hong Kong Polytechnic University (reference number: HSEARS20150109001). The parents were reassured that their participation would not affect the healthcare services that their children received. Informed, written consent was obtained from all of the participants. Childcare services were provided at the request of the parents, so that they could focus on the interviews. In addition, the parents were informed verbally about the aim of the study and about future publications of the study in the scientific literature. All of the information that was obtained was treated as confidential.

### 2.4. Data Management and Analysis

The interviews were recorded and transcribed verbatim in Chinese by two trained student assistants. The first author further checked the transcripts against the audio-taped data for accuracy. Conventional content analysis was used to detect manifest and latent meanings from the data [22,23]. In this process, the audio recordings and transcripts were read several times to obtain a general understanding of the parents’ experiences. Next, different segments of the text were fractured into meaning units and assigned a code. Codes were chosen to retain the core meaning of the participants’ experiences. The codes were then grouped into patterns and labeled under subcategories. The subcategories were grouped into main categories representative of a process of the participants’ experiences over time.

Interpretation followed ideas of metaphorical extraction, which means that the codes, subcategories, and categories drew from the direct meaning of the extracted text (denotation) and, more importantly, from the contextual relationship between the text and the context of the whole interview (connotation) [24]. A further comparison of one part of a participant’s story was made with parts of the stories of other participants, and each interview with all interviews as a whole [24]. Data analysis software was not adopted. Identified codes with a core meaning of the participants’ experiences were extracted from the transcripts and entered into an Excel file for subsequent analysis.

Rigor was achieved through a process of reflexivity and by documenting all analytic decisions, leaving an audit trail. The first author (Y.C.) is a registered nurse in HK, who sought the help of the research team (all authors) to examine her role and search for possible biases throughout the analysis of the data. Notably, all members of the team were nurses with clinical expertise in child and family health. The lead author (Y.C.) and one of the co-authors (Y.M.) are bilingual in English and Chinese. For credibility, initial codes from the first three interviews were translated from Chinese to English and independently coded by the lead author (Y.C.) and an experienced qualitative researcher who is a native speaker of English (D.L.). The translation was carried out by a bilingual professional and was double-checked by the first author (Y.C.) and another qualitative researcher (Y.M.). To ensure plausibility, this co-author (Y.M.), together with the first author (Y.C.), independently scrutinized the codes and sub-categorizations from all of the translated transcripts. Regular meetings of the entire research team (Y.C., D.L, and Y.M) helped to resolve any differences in coding and to refine the analysis to determine the final categories. Last, transferability was attained by comparing the key findings with existing literature about parents of children with asthma and other similar chronic illnesses. Raw excerpts of the data, which exemplify the interpretations, are presented to allow readers to assess how the results may be transferable to their contexts.

## 3. Results

Despite attempts to recruit fathers, a total of 14 parents, who were mothers, participated in the interviews. Two fathers, who refused to participate, reported that they were unfamiliar with their child’s health condition and “unable to provide adequate information” related to their caregiving experiences. One mother left during the interview due to health issues related to her pregnancy.

All of the participants identified themselves as their child’s primary caregiver. Among the participants (age range = 30 to 54 years), two were working mothers and the rest were housewives. Nearly half of the mothers (number of participants (n) = 6) had a personal history of asthma. Four mothers reported that the child’s father (n = 3) or the child’s older brother and sister (n = 1) also had asthma. The children (age range = 3 to 10 years) were diagnosed with asthma at preschool age (range = 6 months to 5 years). As of the interview period, eight of the children suffered from asthma symptoms at least one day per week (see Table 1).

In this study, the parents’ (all mothers) experiences reflected a five category psycho-social process: (1) uncertainty and fear in controlling asthma when asthma was diagnosed; (2) the reoccurrence of the asthma, leading to a search for ways to endure the condition; (3) working through the challenges of controlling asthma; (4) ongoing emotional distress as the asthma continues; and (5) learning to manage asthma better after accumulating experience. Note that symbols and numbers represent different participants (M = mother’s interview number, child’s sex and age).

### 3.1. Uncertainty and Fear in Controlling Asthma When Asthma Was Diagnosed

The participants began to describe the occurrence of asthma attacks when their child was of preschool age (two to three years old) as something that “just happened all of a sudden.” The high-pitched wheezes, which they described as “he he” sounds, together with other flu-like symptoms, recurred with no recognizable patterns. The participants’ uncertainty further intensified when the doctor’s diagnosis was not asthma, as some parents suspected, but “flu” or “bronchial hypersensitivity.” Without a definite diagnosis, the participants found it difficult to make sense of what was happening to their child:

The doctor said that he (the son) didn’t have asthma, but bronchial hypersensitivity, and just delayed the diagnosis. Then, was the so-called ‘bronchial hypersensitivity’ that my child previously had just in fact the same as asthma? I really don’t know.

(M2, son aged 6)

When children began struggling to breathe, and had difficulty speaking, participants realized that this scenario was a medical emergency. They perceived that their child’s life was in danger:

She just wheezed so seriously, and her rib cage was just dented in, just like, losing her breath and saying ‘hah mommy, hah mommy…’ when we (she and the child’s father) were holding her. I was definitely in great fear when we arrived at the hospital. I did not dare to release my arms.

(M6, daughter aged 3)

### 3.2. The Reoccurrence of the Asthma, Leading to a Search for Ways to Endure the Condition

#### 3.2.1. Enduring Suffering during Acute Asthma Attacks

All of the participants reported that their child had visited the emergency department due to an asthma attack at least once during the past six months. Five children required hospitalization for three to five days. According to the hospital’s policy, only one parent, preferably the mother, was allowed to stay with the child during hospital admission. Some of the participants witnessed the efforts of clinicians to control the asthma, such as by administering bronchodilators via aero-chambers or nebulizers, or by using oral suctioning to clear the airway. They reported feeling “heartbroken” and “helpless” when witnessing their child’s struggles. One participant became emotional as she described the scenario:

I was staying outside the curtain and watching how the doctors used the suction tubes to suck the phlegm out and he was shouting ‘Mommy! Mommy! Help me! Help me!’ He was crying so intensely; I was sitting in a chair and just could not stop crying myself. The tears, it was. I indeed felt too heartbroken, it’s a kind of feeling of being stabbed by a knife. I felt that I was useless, helpless too.

(M11, son aged 7)

Many participants described the experience of staying overnight in hospital wards without adequate facilities for rest as “the toughest experience to endure.” A few participants perceived that the treatment offered in the hospital, for example inhalation therapy, was something that they could have done themselves at home. As such, they reported reluctance to go to the hospital again unless they perceived that their child “was on the verge of death.”

#### 3.2.2. Enduring Suffering Whenever Asthma Recurs

Given that a child’s asthma attack could recur unpredictably, the suffering that the participants endured continued. In general, when the participants suspected that there was something “different” about the sound of their child’s breathing, they took a series of prompt actions, such as closely monitoring their child’s breathing, giving symptom-relieving medications, and taking him/her to a nearby clinic for immediate medical advice or to a hospital to prevent life-threatening consequences. Their child’s repeated coughing and wheezing prompted some participants to remain awake all night, to monitor their child’s breathing and to administer the inhaler in a timely manner. One participant said that she had not been able to sleep well for years due to her son’s asthma:

In these three years, when I hear (my child) cough, I will get up at once. I am afraid that he’ll have (an attack) again. The last time (he had an attack), I simply watched my child suffering, and with great fear. He just kept on struggling and crying, so that’s why I have to take care of him for the whole night, and that’s why I cannot sleep well now. Yes, I sleep badly, until now during the night I’ll be in great fear.

(M10, son aged 4)

#### 3.2.3. Distress from Searching for the Reasons for Their Child’s Asthma

Searching for the reason why their child suffered from asthma was important, especially for participants with no family history of asthma. Some of the participants did this by comparing their child with his/her siblings, or reflecting on whether they had paid enough attention to their child’s early development. For one participant, the lack of an explanation for her child’s asthma was a source of great distress:

I was in great psychological distress; (I) wanted to just cry and cry, because why did I give birth to a child to have this (disease)?

(M13, son aged 3)

### 3.3. Working through the Challenges of Controlling Asthma

Many of the participants gave detailed accounts of what they had done to control the asthma, which was very burdensome.

#### 3.3.1. Staying Alert and Preventing Asthma Attacks

All of the participants could list several asthma triggers: second-hand smoke, burning incense, fluffy toys, dust, perfume, cold air, and cold food and drinks. Some of them monitored these triggers, and others firmly prevented their children from coming into contact with them. Furthermore, many of the participants believed that the “blue inhaler” was a life-saving bronchodilator that had to be administered regularly overnight to prevent nocturnal asthma attacks. A few of them were very vigilant using the “blue inhaler.” One participant shared her strategy:

I won’t offer the blue inhaler frequently at one go. First (I’d) try one puff; if he’s okay, then I’d stop. If he’s not that okay, then I’d offer him another puff. I mean, I try to avoid giving too much medication to my child. Then, after one to two minutes, I’d ask him whether he felt that it was easier to breathe.

(M14, son aged 8)

#### 3.3.2. Keep Trying Different Ways to Control a Child’s Asthma

A few of the participants explored different kinds of complementary and alternative dietary therapies, such as “boiled crocodile meat soup” (M6, M7, M9, and M14); “boiled gecko soup” (M7); “boiled fritillary bulbs” (M7, M14), and moxibustion therapy (M14) (a non-invasive procedure that involves burning herbal materials on or above the skin at accu-points to alleviate symptoms). All of these soups or herbs were considered effective over the long term at strengthening a child’s health to improve his/her asthma. As one participant stated:

It’s just like doing homework—you’ve got to do it every single day; otherwise, you can’t see the effect on him (the child).

(M11, son aged 7)

#### 3.3.3. Working through Frustration Due to the Unpredictability of Asthma

Despite the strenuous efforts made by participants to control their child’s asthma, many expressed disappointment when asthma recurred after participants had come to believe that it had “disappeared” (remission of symptoms after a year). Repeated hospital admissions led the participants to lose confidence and to feel powerless. One participant stated:

In the last year I was thinking it should be alright. But then, all of a sudden, it (the asthma) came again not less than a week later, (she) wheezed and was admitted again. Then, (she) had the oral steroids again.

(M4, daughter aged 5)

Another participant reflected that she had “overlooked” the recurrence of asthma:

Asthma is something that I can’t control. Why, when he (her child) got an attack, did it come all over again?

(M3, son aged 10)

#### 3.3.4. Conflicts with Others Surrounding Daily Asthma Care

A few of the participants described their concerns as misunderstood by others. This was particularly common, as their children appeared mostly asymptomatic. They often heard others say: “There’s no need to be that nervous” or “It’s only a cough.”

Misunderstandings were more worrisome when they involved family members. For instance, some of the participants would be questioned by their spouse for hastily seeking medical advice for their child’s flu-like symptoms. Some fathers, who were smokers, might not comply with instructions to keep their child away from tobacco smoke. Further, extended family members were expected to comply with house rules to avoid potential asthma triggers, such as avoiding fried snacks or cold drinks. When these rules were broken, conflict ensued. One participant expressed her resentment:

I told them (the extended family members), please don’t let him (her child) have any junk food or soft drinks, okay? But sometimes they just let my child drink and say something like, ‘Come on, wouldn’t it be too harsh for a kid to not enjoy cold drinks?’ But I said, ‘It’ll be even harsher for me if you attempt to let the child try it once!’

(M9, son aged 5)

#### 3.3.5. Searching for the Best Possible Healthcare Service Suitable for the Family

When a child suffered from an acute asthma attack, most of the participants immediately sought medical help from the emergency department of a public hospital. However, they stated that this did not guarantee that their child would receive immediate treatment. As one participant recalled:

When the triage nurses spot that your child’s oxygen saturation is fine, they obviously won’t act fast.

(M8, daughter aged 8)

On the other hand, some of the participants appreciated the efficiency and comprehensiveness of the medical investigations provided by private hospitals, but were deterred by the cost. One participant stated:

If it’s financially affordable, I’d rather choose a private hospital for my child.

(M6, daughter aged 3)

In addition, finding a pediatric respiratory specialist in HK was challenging. One participant recalled that it was “by luck” that she met a private practitioner who immediately offered inhalation therapy to her child. She further emphasized:

In fact it is really difficult for the parents. First, I didn’t know where I should find (the specialist); second, who is a good one?

(M12, son aged 9)

Deciding on what was “best” was further complicated by differences in the treatment strategies offered by the public and private sectors. For instance, one participant reported inconsistencies in the method of delivering inhaled bronchodilators between the private clinic (via nebulizers, which she had been taught to use) and the public hospital (via aero-chambers). She said that a nurse working in a clinic of a public hospital once told her the following:

As you didn’t really know how to use it (aero-chamber), this led to the fact that he (the child) couldn’t take in all the medication powder.’ But that was not the case.

(M10, son aged 4)

### 3.4. Ongoing Distress as the Asthma Continues

Situations that generated ongoing distress were not at the forefront of the participants’ thoughts, but became prominent as their stories unfolded.

#### 3.4.1. Worries about the Effects on Their Child’s Learning and Development

Most of the participants expressed worry and concern about the potential negative impact of asthma on their child’s learning and development. Specifically, their child’s frequent absence from school was felt to threaten their child’s academic performance and social interactions with peers. Furthermore, many participants were worried about the potential detrimental side effects of the chronic use of inhaled corticosteroids on their child’s development. These include appetite loss, facial puffiness, and impaired growth (M1, M7, M9, M10, M12). In addition, a few participants (M10, M11, M13) questioned whether their child would develop resistance to antibiotics.

#### 3.4.2. Helplessness When Losing the Fight against Asthma

Another concern that was worth noting was the participants’ strong desire to not only control, but to “just get rid of it (the asthma),” given that the asthma symptoms had recurred throughout the years. Those participants believed that they possessed the gene for asthma, and perceived asthma as “something that you must carry for the rest of your life, carrying over to their child’s next generation.” One participant stated:

If you had this disease, you dare not to try to have your own family, right?

(M5, daughter aged 4)

Some of the participants described a few critical moments when they encountered feelings of helplessness and a sense of “being trapped,” or feeling as if “everything was just simply offensive to me.” That moment occurred when the child kept wheezing despite attempts to prevent it, and when the child was too small to voice his/her complaints. One participant projected her anger onto her child in the following way:

Sometimes (I) would project my negative emotions directly to him (the child) and say, ‘Oh! You see, other children are good at all sort of things, but why did this (the asthma) happen to you?

(M3, son aged 10)

#### 3.4.3. Despair Related to Insufficient Support

Of the 14 participants, two recounted feelings of hopelessness and overwhelming distress when living with a child suffering from asthma. Both mothers became emotional and occasionally had to pause during the interview. One of them, who found it difficult to ask for support from others, described her feelings of despair in the following way:

You seem to have no energy every day, but deep in your heart you wish to share everything with others. But you don’t know who you can talk to, as once you share you don’t know how others would regard you.

(M7, daughter aged 6)

The lack of spousal support triggered another participant to contemplate suicide. She recalled:

From the beginning till now, his dad wasn’t involved in anything about the care. (He) simply blamed me for not doing well enough. When she (the child) was two to three years old, she just cried for the entire night. No matter what you did, it just failed. I was not sure what she needed! Every night was the same, and then you definitely had the feeling that (you) wanted to jump from (a) height with the child.

(M4, daughter aged 5)

### 3.5. Learning to Manage the Asthma Better after Accumulating Experience

#### 3.5.1. Asthma Care Becomes Easier When the Asthma Improves

Stressful experiences of childhood asthma care were emphasized by most of the participants. However, some of the participants, who had at least two years of experience caring for a child with asthma, shared positive experiences. These participants became familiar with the nature of the asthma and learned how to differentiate its symptoms from other illnesses. They shared such thoughts as, “I have full confidence now” and “Asthma is not a big deal to me.” In this regard, their emotional distress and the challenges that they faced in caregiving were mitigated by newly formed positive perceptions that came from learning to manage asthma.

#### 3.5.2. Building Support with Others

Multiple sources of support within the hospital, including patient support groups and follow-ups by specialist nurses, were highly valued by the majority of participants. When the responsibility for asthma management was eventually shared with their partners, they expressed relief and were less vigilant about monitoring their children. In addition, peer support helped the participants to ventilate their emotions and concerns. One participant expressed her appreciation of this mutual support:

I felt glad that I have a friend whose son has asthma, as we’re all in the same boat. We chat a lot, and we share (our thoughts and feelings) with each other. It’s a very good way, as we are all up against the (same) difficulty.

(M12, son age 6)

#### 3.5.3. Learning to Live a Normal Life

As the children grew older, their asthma symptoms became less severe. The participants described caring for a child with asthma as being “exactly the same as caring for other ordinary children.” One participant with a 10-year-old son remarked:

You need to take a long time to understand but once you accept the fact that the child has asthma, face it with an open mind and accept it, you’ll feel much happier.

(M3, son aged 10)

## 4. Discussion

To the best of our knowledge, this is one of a few qualitative studies to reveal the unique experiences of HK Chinese mothers in caring for a child with asthma. It reveals that they experience significant psychological distress managing their children’s asthma, which they do in many different ways. Consistent with the findings of other studies [16,25], the mothers of children with asthma in this study expressed feelings of uncertainty and fear when seeking emergency care services, and worried extensively about the recurrence of asthma attacks. They also worried about the impact of asthma on their child’s learning and future development [26], the side effects of medications [27], and the risk of drug dependence [28].

In this study, the mothers stated that a lack of support from their partners exacerbated their distress, which is consistent with findings in other studies of HK Chinese families rearing children with chronic and behavioral health problems, such as eczema [28], autism [29], and attention deficit hyperactivity disorder [30]. Moreover, as reported elsewhere, the mothers in this study mentioned that conflicts with family members arose over the management of the asthma, such as when family members held different perceptions of how to handle the asthma symptoms, and when there were disagreements over the asthma management routines at home [31,32].

However, in contrast with the findings of other studies, the distress of the HK Chinese mothers in this study was not necessarily resolved with emergency medical care for their child’s asthma attacks [5], nor from being involved in treatment decisions [33]. Rather, some mothers reported continued expressions of helplessness and self-blame for long periods of time. This process was marked by a tendency to negatively evaluate their role as parents, the asthma prognosis, the adequacy of family support, and the adequacy of the healthcare system as a whole. In particular, mothers’ perceptions of guilt appeared more pronounced and prolonged, as compared to parents of children with asthma in other studies [6,34,35]. We posit that Hong Kong parents may define the problem of asthma differently, and choose multiple behavioral options, due in part to an implicit cultural belief that their efforts might “cure” the asthma one day.

In the present study, the mothers applied various strategies to control their child’s asthma, such as searching for the root causes of the asthma; avoiding environmental triggers of the asthma; using a “trial-and-error” approach to find the most appropriate healthcare service, similar to the “doctor shopping” found in many countries [36]; and maintaining heightened vigilance of their child’s health condition. While these control strategies worked in the short term, the mothers often could not fulfill their desire to control the asthma permanently due to its unpredictability and recurrence, and perhaps also to the persistent belief that they ought to be able to control or “cure it.” Hence, this desire may have exacerbated their psychological distress and created the sense that they were faced with an overwhelming crisis, which led a few mothers to report feelings of extreme despair with suicidal ideation early in the process of adapting to their child’s asthma.

In fact, the mothers in the interviews might had gone through the process of excessively evaluating unwanted emotional experiences and making deliberate efforts to control or escape such experiences. This may have led to greater dysfunction (e.g., managing childhood asthma ineffectively) and increased distress. Indeed, self-blame can be regarded as a maladaptive emotional control strategy that allows individuals to avoid difficult thoughts and feelings when a stressful event occurs [37].

This study has several limitations. One of the major limitations was that no fathers could be recruited in our study. In particular, we found that two fathers refused to participate in our interviews because they felt that they were “unable to provide adequate information” related to their caregiving experiences. In this regard, fathers may tend to devalue the importance of their involvement in childcare when compared to mothers [38]. Additionally, in Hong Kong, Chinese parents tend to conform to conventional gender roles, such that mothers, rather than fathers, are perceived to be the major caregivers for their children [39]. Together, this may have created a tendency for mothers, rather than fathers, to voluntarily participate in our study, making it difficult to recruit fathers.

The challenge of recruiting fathers to share their caregiving experiences has been discussed in the literature [40]. Goldstein and colleagues suggested that mothers are often the primary caregivers of children with chronic health conditions, and are usually the ones who accompany their children to seek healthcare services. Hence, it is easier to gain access to mothers rather than fathers for interviews in healthcare settings [40]. Goldstein and colleagues further posited that fathers may regard interviews as a form of seeking social support, which may deter them from voluntarily participating in them [40].

In Western studies that explored the experiences of parents caring for a child with a long-term condition, fathers remain under-represented [41]. This under-representation is also commonly found in studies conducted in HK [30,42]. Indeed, a review conducted by Goldstein and colleagues [40] found that over the past ten years, only one study, conducted by Cashin and colleagues (2008), explicitly explored paternal experiences in childhood asthma management, and that study showed that fathers actually felt relieved once their child’s diagnosis of asthma was confirmed [43]. During the post-diagnostic phase, the fathers’ caregiving experiences often included some degree of both a sense of mastery of their child’s health problem and a feeling of uncertainty [43]. In future work exploring parental caregiving experiences, more effort will be needed to ensure that fathers are better represented.

In addition, we acknowledge that the diversity of the parents’ experiences in childhood asthma management in the present study is limited, as the recruitment of participants was conducted in one public hospital in HK. This was to establish a homogeneous group of parents, whose children would have at least one experience of being treated by emergency care services due to a life-threatening asthma attack. Hence, the caregiving experience reported by the mothers in this study may not be transferable to other parents of children with less severe asthma.

Despite these limitations, this study adds an empathetic and detailed understanding of the experiences of HK Chinese parents, especially typical mothers, who are caring for a child with asthma. In particular, this study has implications for clinical practice, revealing that healthcare providers should demonstrate more empathy and attention to parents with young asthmatic children. Structured asthma education programs that offer adequate information on asthma treatment and skills training, in addition to the management of symptoms, might be helpful to parents [44,45]. More importantly, this study indicates a pressing need for psychological support. Psychological interventions that place a major emphasis on developing acceptance and mindfulness, such as Acceptance and Commitment Therapy [46] and mindfulness-based therapies [47], may be able to support parents. More mixed-method investigations are warranted to understand the accounts of parents, as well as to measure their psychological processes, when managing their child’s asthma.

## 5. Conclusions

In summary, this study highlights the substantial psychological distress experienced by HK Chinese parents, especially typical mothers, in caring for a child suffering from asthma. Such distress was prominent when encountering an asthma crisis or when asthma symptoms recurred; further exacerbating their desire to take control over the asthma. Considering the desire of parents to achieve a cure for their children’s asthma, and the unpredictability of asthmatic crises, helping parents to understand their limits of control may enable them to cope better. The findings of this study suggest that addressing the psychosocial needs of parents may help them to better manage their children’s asthma condition.

## Figures and Tables

**Table 1:** Characteristics of the interviewed parents and their children.

Parent’s Information				Child’s Information			
Participant	Relationship with the Child	History of Asthma	Other Family Members’ History of Asthma	Gender, Age	Age of Asthma Diagnosis	Current Use of ICS for at Least 6 Months	Average Number of Days with Asthma Symptoms per Week in the Past 30 Days
01	Mother	No	Child’s elder brother and sister	Male, 4	2	No	No symptoms
02	Mother	No	Father	Male, 6	5	Yes	1 day with symptoms
03	Mother	No	No	Male, 10	4	Yes	1 day with symptoms
04	Mother	No	No	Female, 5	2	Yes	5 days and 5 nights with symptoms, 5 days requiring reliever therapy
05	Mother	Yes	No	Female, 4	3	No	No symptoms
06	Mother	Yes	Father	Female, 3	2	Yes	2 days with symptoms, 2 days requiring reliever therapy
07	Mother	Yes	No	Female, 6	2	Yes	No symptoms
08	Mother	No	No	Female, 8	2	Yes	No symptoms
09	Mother	Yes	No	Male, 5	2	Yes	No symptoms
10	Mother	No	Father	Male, 4	1	Yes	4 days and 4 nights with symptoms, 4 days requiring reliever therapy
11	Mother	No	No	Male, 7	1	Yes	2 days with symptoms, 2 days requiring reliever therapy
12	Mother	Yes	No	Male, 9	3	Yes	1 day with symptoms, 1 day requiring reliever therapy
13	Mother	No	No	Male, 3	3	Yes	No symptoms
14	Mother	Yes	No	Male, 8	3	Yes	2 nights with symptoms, 2 nights requiring reliever therapy

Note: ICS = inhaled corticosteroid. Reliever therapy refers to inhaled short-acting bronchodilators for quick relief of the child’s asthma symptoms.
